# Investigating population-level immunosenescence: From bench to bedside

**DOI:** 10.3389/fimmu.2022.949928

**Published:** 2022-08-17

**Authors:** Lucas Leite Cunha, Victor Alexandre dos Santos Valsecchi, Laura Sterian Ward

**Affiliations:** ^1^ Laboratory of Molecular and Translational Endocrinology, Division of Endocrinology, Federal University of São Paulo, São Paulo, Brazil; ^2^ Discipline of Internal Medicine and Laboratory Medicine, Federal University of São Paulo, São Paulo, Brazil; ^3^ Laboratory of Cancer Molecular Genetics, Faculty of Medical Sciences, University of Campinas (Unicamp), Campinas, Brazil

**Keywords:** immunosenescence, inflammaging, population, adaptive immunity, innate immunity

## Abstract

The immune response is remodeled with aging in a process called immunosenescence. Some immunologists conceive immunosenescence as an adaptation of immunity to the aged immune-environment rather than a merely collapsed reactivity of immune cells against microbes and tumor cells. Others believe on an uninterrupted activation of the innate immune system with aging, leading to a low grade, sterile and chronic proinflammatory state called inflammaging. For instance, it is possible that chronic infection by cytomegalovirus leads to persistent production of viral load. This phenomenon offers periodic stimuli to the immune system that ultimately contribute to the remodeling of the immune response. If investigating immunosenescence at the cellular level is already a difficult task, considering the population level is much more complex. However, by studying immunosenescence at the population level, we can extract valuable results with viable applications. While studies with animal models allow scientists to deepen their understanding of the mechanisms of immunosenescence, studying large populations can bring practical innovations to medicine and the health system. Many researchers and funders have dedicated themselves to producing methods for the evaluation of immunosenescence on a large scale, aiming to elucidate new mechanisms by which diseases are established in the elderly. The description of how the immune response is remodeled with aging emerges as a new tool to identify the subset of subjects in which unhealthy aging is a matter of time, to help better individualize clinical management and select patients who may benefit. of early interventions. This review focuses on functional assays as valuable methods for measuring the remodeling of the immune response with aging and discuss their clinical impact. We also recall fundamental concepts for understanding the aging process of the immune response. In addition, we highlight future prospects for immunosenescence research.

## Introduction

The increase in maximum life expectancy that humanity has experienced in recent decades has raised concerns related to healthy aging. In this scenario, understanding the modifications that occur in the immune system emerges as a milestone for physicians and scientists. The immune response is fluidly altered with aging (immunosenescence) and results in increased susceptibility to several clinical conditions, such as infectious diseases (1). This essentially negative concept has given way to another broader and evolutionary-based concept. Some immunologists conceive immunosenescence as an adaptation of immunity to the aged immune-environment rather than a collapsed reactivity of immune cells against microbes and tumor cells. Some of these modifications have been shown to have a negative impact on the functioning of the various immune system components and may have a significant impact on patients’ responsiveness to infections, vaccination and diminished immunosurveillance (2).

**Figure 1:**
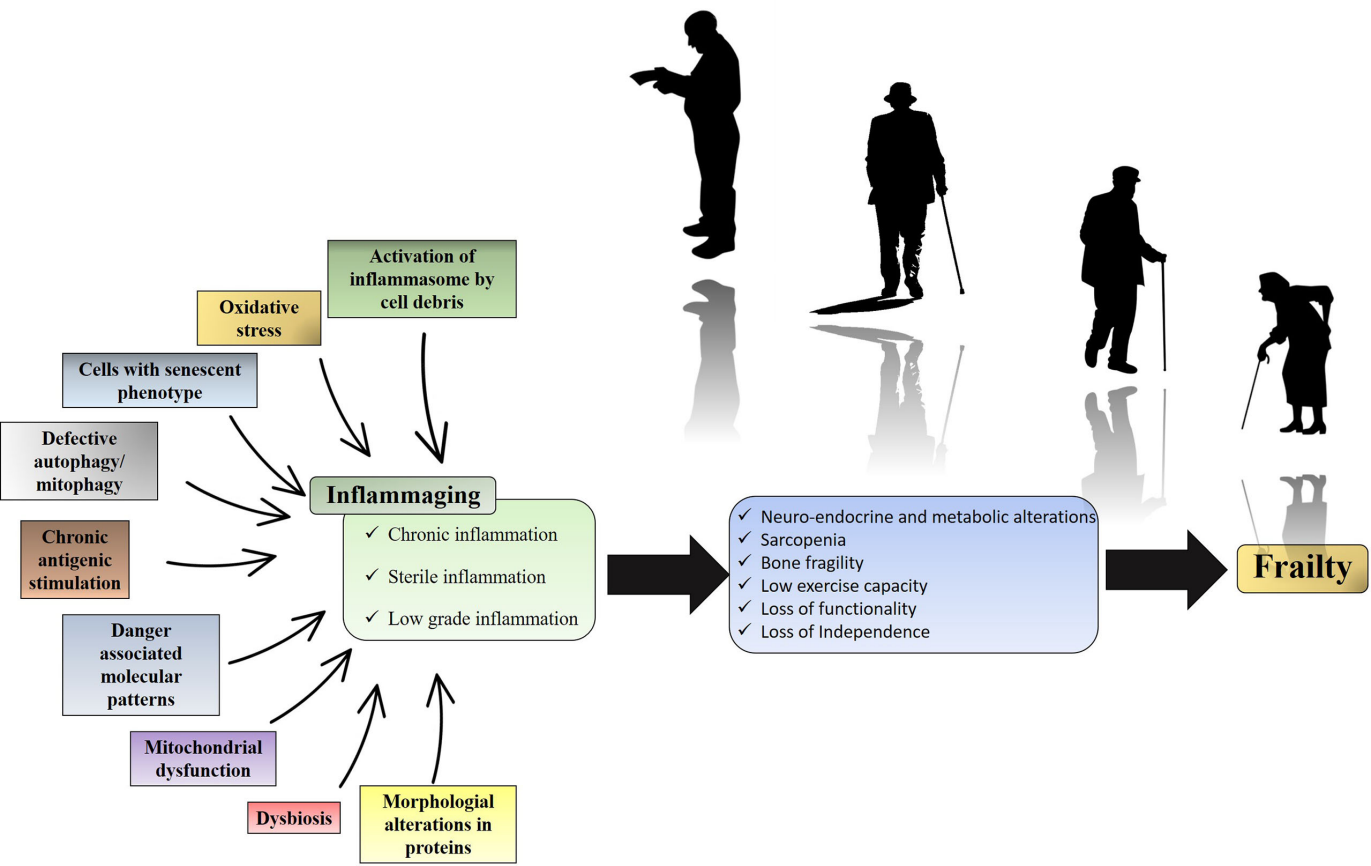
Inflammaging and its clinical impact. Over time, endogenous or exogenous chronic immune stimulation leads to an increase in the pro-inflammatory tone characteristic of inflammaging. The metabolic consequence of this pro-inflammatory state is the biochemical imbalance that culminates in loss of strength, loss of performance and loss of functionality. While the figure summarizes the impact of inflammation on frailty, it is important to note that frailty is also influenced by sociodemographic risk factors.

Both adaptive and innate immunity are influenced by aging. Some subjects experience uninterrupted activation of the innate immune system, leading to a relative increase in activating cytokines and the production of innate cells. Once uncontrolled, the unproportioned activation of innate immunity may be detrimental and associated with a decrease in functionality in a clinical syndrome known as frailty (**Figure 1**) (3). The pro-inflammatory state occasioned by continuous immune stimulation is called inflammaging (3). The source of this stimulation may be endogenous or exogenous (4–8) and its final product is a low grade, sterile and chronic pro-inflammatory state (3). Inflammaging may disrupt neuro-endocrine and metabolic homeostasis, leading to loss of lean mass and low performance among elderly (9). It is important to highlight that chronic low-grade inflammation is relevant, but not sufficient to lead to frailty. The risk for this multifactorial condition can be influenced by several sociodemographic variables (10).

Likewise, adaptive immunity remarkably changes as age evolves. Bone marrow is reorganized, and the hematopoietic stem cell pool most prominently differentiates into the myeloid lineage, outnumbering the lymphoid compartment (11). To this progenitor disequilibrium should be added thymic involution, which compromises the generation of new naïve T cells. However, the assessment of the functionality of the immune system rather than the absolute number of immune cells alone seems to better reflect the complexity of reshaping process of the immune response with age.

Assessing the functionality of the immune system is a great challenge. In fact, many functional assays have been developed to trace the steps related to the establishment of the immune response. Herein, we discuss the assessment of immunosenescence in human populations. The literature was accessed through international databases where we searched for articles on clinical and experimental research that added new knowledge about the field of immunosenescence. Our aim is to focus on functional assays as valuable methods to measure immune response remodeling with aging and their clinical impact. We discuss fundamental concepts for understanding the aging process of the immune response and present future perspectives in the investigation of immunosenescence. This discussion will bring new insights to the clinical management of the elderly.

## Physiology of immunosenescence

Aging is associated with changing patterns in physiological functions. More than a simple state of relative immunodeficiency, immunosenescence must be considered a complex ongoing remodeling of immune cells and their biological microenvironment. Indeed, immunosenescence has been recognized as a more intricate process involving the composition, phenotype and function of cells from both innate and adaptive subsets of immunity (12, 13). **Table 1** summarizes the physiological alterations that occur with immune cells.

**Table 1:** Physiologic alterations that immune cells face with aging.

Cell	Type of immune response	Features acquired with aging	Clinical impact	Reference
**NK**	Innate	✔ Impaired perforin degranulation	✔ Slow wound healing ✔ Immunesurveillance and tumorigenesis	(14)
**Neutrophil**	Innate	✔ Impaired chemotaxis ✔ Impaired cytotoxic function	✔ Slow would healing ✔ Immune response against infectious diseases	(15–18)
**Macrophage**	Innate	✔ Impaired phagocytosis ✔ Impaired cytokine secretion ✔ Impaired antigen presentation ✔ Impaired effector mechanism of immune response	✔ Slow would healing ✔ Immune response against infectious diseases	(15)
**B cell**	Adaptive	✔ The repertoire diversity of B cells reduce over time ✔ Impaired class switch recombination	✔ Vaccine response ✔ Antibody secretion	(19–21)
**T cell**	Adaptive	✔ Impaired development of new naïve cells ✔ Impaired secretion of cytokines by CD4+ cells ✔ Impaired cytotoxic function of CD8+ cells	✔ Vaccine response ✔ Antibody secretion ✔ Immune response against virus	(22, 23)
**Dendritic cell**	Adaptive	✔ Impaired antigen presentation ✔ Diminished IFN-gamma production	✔ Vaccine response ✔ Antibody secretion ✔ Immune response against infectious diseases	(15)

Age-related remodeling may influence the homeostasis of neutrophils, natural killer (NK) cells, monocytes/macrophages, and dendritic cells, all considered hallmarks of the innate immune response (15). It has been recently reported that the function of innate immunity extends beyond protection against infections (14, 24). NK cells also promote granule exocytosis targeting senescent cells (24). NK cells from elderly individuals exhibit impaired perforin release upon stimulation (14), suggesting that NK cell immunosenescence may be a mechanism that justifies the accumulation of senescent cells in aged tissue. It is noteworthy that human aging is associated with a reduced frequency of NKp46+ NK cells (25). The NKp46 receptor mediates the recognition and elimination of inflammatory cells by NK cells (14). This reinforces the notion that the rates of NK-cell-mediated inflammatory cell apoptosis may be reduced in elderly individuals, explaining the slower resolution of inflammation (26).

The age-related remodeling of polymorphonuclear leukocytes and macrophages may help to explain the delay in wound healing also observed in older patients (16). In addition, aging is associated with a subclinical chronic inflammatory state characterized by elevated levels of proinflammatory cytokines and acute phase proteins, as well as reduced levels of anti-inflammatory cytokines, a state called inflammaging (27). Most likely, elderly individuals sustain a low-grade inflammation by stable antigenic stimulation (4). The source of antigens may be exogenous, such as cytomegalovirus (5, 6), or endogenous, such as posttranslational-modified macromolecules (7).

**Figure 2:**
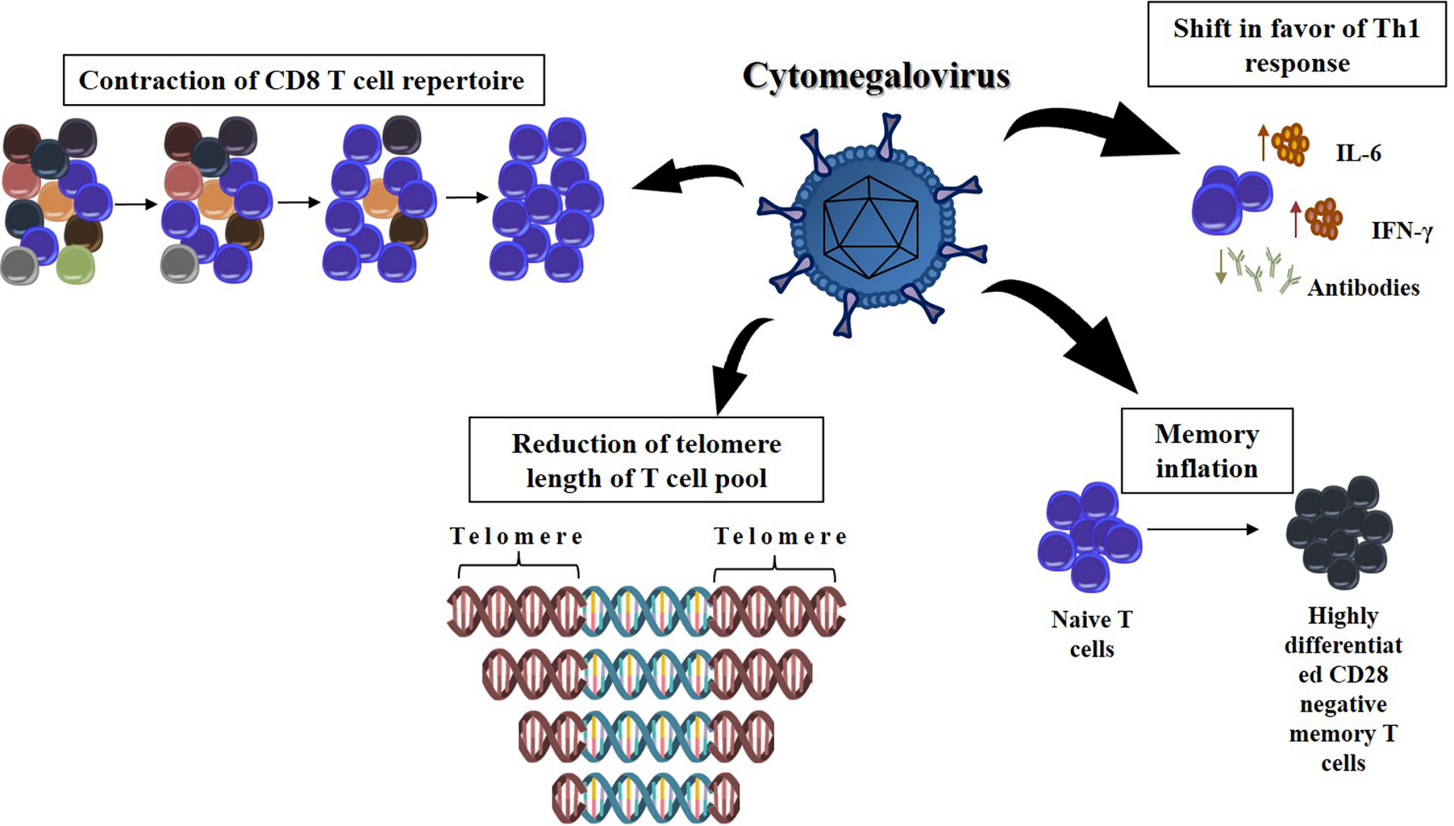
The effect of chronic infection by cytomegalovirus at the remodeling of the immune response with age. The CMV infection can dramatically decrease the T cell repertoire, narrowing the range of new pathogenic bioagents that can be recognized by the immune response. Furthermore, the presence of CMV DNA in peripheral blood monocytes was longitudinally associated with higher serum levels of IL-6, suggesting shift in favor of the Th1 immune response. Each cycle of viral reactivation generates a subset of CMV-specific CD8+ T cells. It causes these terminally differentiated lymphocytes to be overrepresented in immune system (memory inflation). It is also possible that T cells chronically infected with CMV have shortened telomeres, which limits the lifetime these cells are available for an immune response.

Cytomegalovirus (CMV) is a beta herpesvirus that completes its cycle in human cells and can be in latency (reservoir cells include hematopoietic progenitors, monocytes, dendritic cells, endothelial cells, and lymphoid vessels) for a long time (28). **Figure 2** shows the many steps through which CMV can influence the remodeling of the immune system over time. The first observations linking CMV infection to immunosenescence were reported by Looney et al, who demonstrated that there is a strong and independent association between CMV seropositivity and an increased number of CD28- CD4 or CD8 T cells (29). After an acute infection, CMV elicits a CD8-based T-cell response, as well as CD4 and B lymphocyte activation (30). Persistent production of CMV viral load offers periodic stimuli to the immune system, leading to the maintenance of virus-specific T cells in lymphoid organs and peripheral vessels (31). Thereafter, a subset of CMV-specific CD8+ T cells is generated in each viral reactivation cycle (a phenomenon known as memory inflation) (31, 32). The majority of CMV-specific CD8+ T cells are mature and terminally differentiated (33), and CMV infection is associated with a reduction in the telomere length of the circulating T-cell pool (34). Both these features are typical of immunosenescence. Interestingly, Khan et al. demonstrated that in elderly individuals, CMV positivity leads to the development of oligoclonal populations of CMV-specific CD8 T cells that can constitute up to one-quarter of the total CD8 T-cell population (35). These results suggest that CMV infection could contribute to the acceleration of immunosenescence by promoting the contraction of the CD8 T-cell repertoire with aging. However, there are some contradictory results that indicated no causality with inflammation (36). Some authors failed to show the association between CMV and frailty, and CMV infection was even correlated with improved survival in the elderly, indicating that further investigation is needed to clarify the role of CMV in immunosenescence (37, 38).

The adaptive immune response also changes as age evolves. The adaptive immune response may become impaired with age as a consequence of thymic involution and insufficiency of hematopoietic stem cells (39, 40). The elderly are less able to respond to neoantigens than young individuals because fewer new naïve cells leave the thymus in elderly individuals, although some older people may experience a phenomenon of partial replenishment of the T cell repertoire (22, 23). Indeed, T cells are the most affected compartment of the adaptive immunity response because T cells from aged individuals are usually lower in number and slower than those of young individuals regarding proliferation, telomerase activity, or induction of signaling events (41, 42). Furthermore, almost all adult regulatory T lymphocytes are suppressive and apoptosis-prone populations at a late stage of differentiation (43). In addition, transplant patients at old age may present increased incidence and severity of rejection, as well as increased resistance to tolerance induction due to accumulation of both T and B memory cells (44, 45).

It is suggested that naïve T and B cells become dysfunctional with aging. In contrast, the function of memory T and B cells is relatively maintained (46–48). This can be explained by intrinsic dysfunctions in which T and B cells accumulate over time. When naïve T lymphocytes collected from elderly individuals are stimulated *in vitro*, they show slower and fainted activation than those obtained from younger individuals (49, 50). The decline in T lymphocyte activation with age may be partially explained by impaired immune synapse construction (51), weakening of the signal transduced into the cytoplasm (52), dysregulation of cytoskeletal function (53), modification in the glycosylation pattern of molecules considered essential for the activation of T cells (54) and insufficient production of IL-2 following T-cell activation (55).

The B cell response in the elderly has intrinsic age-dependent dysfunction. Investigations of vaccine trials suggest that the diversity of the B cell repertoire decreases over time, particularly in frail patients (19, 20). Elderly individuals are deficient in class switch recombination, an immunological phenomenon in which B cells secrete specific antibodies with different effector functions from different classes (21). Class switching is well characterized in the germinal centers of lymph nodes and spleen and can be facilitated by both T-dependent and T-independent stimuli (21, 56). Activation-induced cytidine deaminase is known to induce class switch recombination and immunoglobulin somatic hypermutation (57). Activated old murine B cells also have less activation-induced cytidine deaminase and fewer antibodies exchanged, reinforcing that the effectiveness of humoral immunity is impaired with aging (58, 59).

## Functional assessment of immunosenescence

The functional assessment of the aging of the immune system may be of clinical relevance. In fact, some elderly experience some clinical symptoms and signs that suggest a mild impairment of the immune response. Clinicians must be aware of certain patterns such as recurrent infections, chronic diarrhea, malabsorption and coexistence of infection, autoimmunity and malignancy (60). The management of elderly people with possible deficiency in the immune response must include the distinction between a primary and a secondary immunodeficiency (61). Secondary causes (eg, poorly-controlled medical conditions, malnutrition, malignancy, drug induced immunossupression, metabolic diseases, HIV) are more common among older adults (62). After the initial tests for common clinical conditions, the immune response may be assessed by laboratory techniques such as serum immunoglobulins measurement and lymphocyte subsets *via* flow cytometry (63). In addition, there is some future perspective on the assessment of cytokine production assays that may reveal specific immune defects among adults suspect for immunodeficiency (64).

The functional assessment of the immunosenescence is relevant at a research level. The physiological process leading to immunosenescence essentially begins at the molecular and cellular levels. Many of the alterations responsible for the remodeling of the immune system can be scrutinized through methods that could indirectly reflect the aging of the immune cells. Naturally, none of these methods is flawless, and it is up to the scientist to discern which cellular aspect of immunosenescence is better represented by a specific method. **Table 2** lists the main pathways used to assess immune function with age.

**Table 2:** The assessment of the immune response with aging.

Methods of assessment of immune response	Description of the function and remodeling with aging	Clinical impact or potential to clinic	Reference
**Immunephenotype by flow cytometry**	Assess the number of immune cells, phenotypic diversity, the immune repertoire and biomarkers of immune activation.	Allow the description of the pool of different population of immune cell.	(65)
**Mice model of premature immunosenescence**	Guayerbas et al. described a model of premature immunosenescence in mice, based on the demonstration of premature decline in both immune parameters and the behavioral tests in Swiss outbred mice. The mice model of premature immunosenescence was refined and new other models was developed as well.	Mice model allow the investigations of mechanisms of immunesenesence. In addition, it allow preclinical test with molecules that may target immunesenesence mechanisms.	(66–68)
**Assess the neutrophil extracellular trap (NET)**	NET is the extracellular release of granule proteins bound to a decondensed chromatin meshwork to eliminate pathogen. NET formation can be assessed by immunefluorescence. NET formation is impaired with aging.	Impaired NET formation may increase susceptibility to bacterial infection.	(18, 26, 69, 70)
**Dosage of reactive oxygen species**	Free radicals by polymorphonuclear leucocyte of older adults was decreased under appropriate stimulation.	Impaired free radical formation may decrease of microbicidal potential of innate immune cells.	(71, 72)
**T cell exhaustion**	The constant antigen stimulation progressively exhausts the T-cell, which gradually loss his proliferative and responsiveness capacities. Exhausted and immunosenescent T-cells are generated by exposure to many different antigens over a lifespan, such as CMV	The hallmarks of T cell exhaustion may be potential target to immunetherapy	(52, 73–77)
**Single-cell network profiling**	Uses multiparameter flow cytometry and it monitors phospho-protein responses to molecular stimuli at the single cell level. Major of age-associated immune signaling nodes occurred within naïve cells, which functionality has been reported to be remodeled over time	Allow to monitor the functionality of the immune response with aging. Potential target for reverting T helper cell immunosenescence	(22, 55, 78, 79)
**Stimulated cytokine signaling, production and secretion**	The method allow the phospho-protein analysis of cytokine signaling, cytokine production and gene expression from stimulated PBMC in a stimulation-response way. It analyses the immune response through different paths, integrating cellular, protein and genomic data in a population level. Phospho-proteins assessment was strongly dependent of CMV status. Stimulated cytokine secretion was associated with age and phenotype of immune cells was associated with both CMV status and age.	It allow the mitigation of the idea that one single analyte of immune system could solely enclose the immune age likewise an “immune clock”	(80)

Neutrophils are the most prevalent cells among white blood cells. Neutrophils are indispensable cells of the innate immune response, as they act as a fierce trigger of various immune effector mechanisms. One of these mechanisms is the formation of the extracellular neutrophil trap (NET), previously described as the extracellular release of granular proteins linked to a decondensed chromatin mesh that favors the elimination of microbes and parasites (81, 82). As a potent effector mechanism, it must be strictly regulated by the immune system (83). When disturbed, NET formation could result in vascular damage and tissue insult characteristic of autoimmunity (84, 85).

The microbicidal potential of neutrophils is reduced with age (17, 18). A decline in NET generation, degranulation and phagocytosis over time (69, 70) has been documented in both animal models (86) and humans (18). Hazeldine et al. were the first to assess NET formation in the context of immunosenescence. They observed that older adults had a lower ability to generate NET as significantly lower amounts of extracellular DNA were extruded by neutrophils treated with IL-8 or LPS, probably due to impaired signal transduction following IL-8 and LPS stimulation (18).

Reactive oxygen species (ROS) are free radicals fundamental for the microbicidal function of innate immune cells (87). ROS not only directly contributes to bacterial elimination, but can also trigger NET formation (88, 89). The production of free radicals by polymorphonuclear leukocytes has been investigated, and most studies found that the production of free radicals by properly stimulated polymorphonuclear leukocytes was decreased in the elderly (71, 72).

The assessment of immunosenescence at the cellular level has been widely available through the characterization of animal models of premature immunosenescence. Mice with the earlier immunosenescence phenotype (66), when longitudinally investigated, have a shorter life span than control mice. Their peritoneal leukocytes exhibit lower proliferative response to stimuli, a decline in NK activity and increased TNF alpha production compared to control mice (66). In addition, macrophages of premature models are less functional, with a marked loss of antimicrobial capacity (90).

New improved models of immunosenescence have been developed (67, 68). They allowed us to observe that immunosenescence is accompanied by complex neuroimmunoendocrine reshaping that is expressed by neuropsychological deficits, poor neuromuscular coordination, and worse sensorimotor abilities (66, 68, 90). The key phenomenon that seems to be central is oxidative and inflammatory stress, which, not without reason, are associated with several chronic non-communicable diseases prevalent in the elderly (40, 67, 91). Indeed, immune cells harvested from the spleen and thymus of immunosensitive preterm mouse models had lower values of antioxidant defenses and higher values of oxidants and pro-inflammatory cytokines than cells from controls (67). Interestingly, the antioxidant versus oxidant balance of immunosenescent preterm mice was similar to that of cells from aged animals, suggesting a causal relationship between this imbalance and the remodeling of the immune response observed with aging (67, 92).

T-cell function declines with aging, as described above (3). It is noteworthy that the T cell dysfunction observed in the elderly is not the same concept as the dysfunction reported as T cell exhaustion. Indeed, T cell exhaustion is the least responsive state mediated by conditions such as chronic viral infection and cancer (73), as exemplified in **Figure 1**. Constant antigenic stimulation progressively depletes the T cell, which gradually loses its proliferative and responsive capabilities. Chronic viral infection is an example of chronic stimulation. Persistent exposure to viral particles leads to upregulation of co-inhibitory (93). The co-inhibitory molecules downregulate the TCR-stimulated intracellular signal and T cells become anergic (94). Interestingly, while different in concept, the T-cell dysfunction observed in immunosenescence shares many mechanisms with T-cell exhaustion.

Investigations using a mouse model suggest that there is an accumulation of T cells with an exhausted phenotype over time (52, 74–77). In fact, Shimada et al. demonstrated that both the mRNA and protein expression levels of PD-1 and CTLA-4 are higher in cells from old mice than in cells from young controls (74). The majority of PD-1-positive cells were not activated and had an effector memory phenotype (74). When challenged with anti-CD3 and anti-CD28 antibodies, these PD-1+ T cells failed to proliferate, suggesting that this subset of cells from old mice was hypo responsive (74). Investigations focused on chronic viral infections revealed that Tim-3 is another coinhibitory receptor marker of exhausted T cells (95, 96). Tim-3 interacts with Galectin-9 and leads to T-cell death (97). Lee et al. also investigated T-cell exhaustion in old mice and reported an accumulation of both Tim-3-positive and PD-1-positive T cells with aging (76). The proliferative capacity of both Tim-3-negative PD-1-positive and Tim-3-positive PD-1-positive CD8 T cells was impaired, reinforcing that these immunosenescent and exhausted cells display an anergic phenotype (76). Interestingly, the age-related exhaustion observed by Lee was not exactly the same as that classically reported in chronic infection-induced exhaustion, in which abundant CD160 expression was noted (98). The authors also noted that age-associated Tim-3-positive PD-1-positive CD8 T cells secrete high levels of IL-10 and have the potential to stimulate the expression of IL-10 in normal CD8 T cells, contributing to the increased systemic levels of IL-10 (76). According to Lee´s results, it is reasonable to consider that exhausted and immunosenescent T cells are generated by exposure to many different antigens over a lifespan, rather than by a single specific viral infection (76). This concept is consistent with the previous thought that conceive CMV is one of the triggers of the immunosenescent phenotype.

Song et al. described that immunosenescent T cells from old adults are enriched with TIGIT-positive CD8 T cells (77). T-cell immunoglobulin and immunoreceptor tyrosine-based inhibitory motif (ITIM) domain (TIGIT) are coinhibitory receptors expressed on activated T cells and compete with their costimulatory counterpart CD226 for the same ligands (CD155 and CD112) (99, 100). This close interaction leads to immune suppression in models of tumors and chronic infections (99, 100). Older adults may accumulate TIGIT-positive T cells, which exhibit a terminally differentiated, depleted phenotype (77). TIGIT-positive CD8 T cells from elderly individuals have been shown to retain their proliferative capacity while TNF-alpha, IFN-gamma, and IL-2 are poorly produced when compared to TIGIT-negative CD8 T cells (77). In addition to this dysfunctional feature, exhausted and immunosenescent TIGIT-positive CD8 T cells obtained from the elderly are more susceptible to cell death (77), suggesting that although T cell exhaustion and immunosenescence are different phenomena, they may be different points on the same spectrum of remodeling immunity.

## Assessment of immunosenescence at the population level

If investigating immunosenescence at the cellular level is already a difficult task, considering the population level is much more complex. However, by studying immunosenescence at the population level, we can extract valuable results with viable applications. The inference of an immunological age, for example, is only possible by studying human populations. While studies with animal models allow scientists to deepen knowledge of immunosenescence mechanisms, studying large populations can bring practical innovations to medicine and the health care system. It is no wonder that many researchers and funders have devoted themselves to producing methods for the assessment of immunosenescence on a large scale.

Immunogerontological studies have expanded over the last three decades, mainly after the publication of the SENIEUR protocol. The SENIEUR protocol was developed by Ligthart et al. in a working party in the framework of the EURAGE Concerted Action Programme on Aging of the European Community and was designed to provide a reference measurement of immunosenescence in the healthy aged population (101). It consists of the establishment of strict admission criteria for immunogerontological studies, intending to avoid selection bias. This made it possible to conduct investigations that identified that a loss of T-cell homeostasis takes place as the age goes on (102). It was reflected by a decrease in the number of CD4 cells, an increase in the CD8 subset in individuals with an inverted CD4:CD8 ratio, and proliferation of terminally differentiated effector memory T cells (102, 103). In addition, CMV DNA in peripheral monocytes was longitudinally associated with serum IL-6 levels (104). In fact, CMV-dependent T-cell immunosenescence affects healthy aging and may be related to frailty, loss of functionality, morbidity and mortality (105–108). This finding reinforces the idea that immunological age is determined not only by endogenous but also by exogenous factors.

To perform a more real-world characterization of immunosenescence, Nilsson et al. used a SENIEUR-modified protocol to include elderly individuals in the investigation of immune parameters (109). The design of the study allowed them to compare different subgroups defined according to health status (very healthy, moderately healthy, and frail groups). Comparison between the elderly and middle-aged groups suggested an increase in the subset of cells with the immune risk phenotype in the elderly, characterized by both a high CD8 and low CD4 proportion and poor T-cell proliferation in peripheral blood lymphocytes. Interestingly, this difference was independent of the health status, suggesting that phenotypic characterization alone fails to predict healthy aging.

To skew the reductionist approach in the analysis of individual components of the immune system, Longo et al. described a new technology known as single-cell network profiling (SCNP) (78). Single-cell network profiling uses multiparameter flow cytometry and monitors phospho-protein responses to molecular stimuli at the single cell level. By exposing immune cell signaling networks to different inputs, SCNP can discern unique immune cell responses, assessing the signal produced by a phospho-protein mediator. The SCNP requires the definition of the “signaling node” to refer to a specific protein readout in the presence of a given stimulus. Then, the response to IL-4 stimulation can be assessed using p-STAT5 as a readout. Since each signaling pathway is measured in each cell subset, the cell subset is noted as follows, e.g., “IL-4 → p-STAT5 | T helper lymphocytes”. The authors observed the impact of age on the immune signaling responses of four nodes (negative correlation for IFN-α → p-STAT5 | CD45RA+ cytotoxic T cells; negative correlation for IL-27 → p-STAT5 | CD45RA+ cytotoxic T cells; negative correlation for IL-4 → p-STAT6 | CD45RA+ cytotoxic T cells; and positive correlation for IL-2 → p-STAT5 | CD45RA+ Th cells). Interestingly, all age-associated immune signaling nodes occurred within CD45RA+ T (naïve) cells, whose functionality has been reported to be remodeled over time (22). The authors raised an important consideration by noting that only a single age-dependent node involved CD45RA+ helper T cells, which was an increased activation of Stat 5 induced by IL-2. This signaling is necessary for lymphocyte activation and decreases with aging, suggesting that it could be a potential target to reverse helper T-cell immunosenescence (55). It is noteworthy that few elderly people were included in the study, which may make it difficult to extrapolate these results as a model of immunosenescence.

The same group performed the functional analysis of immune cells in an independent cohort and obtained blood samples from 174 healthy individuals (144 over 65 years and 30 between 25 and 40 years) (79). They used a similar approach to the SCNP technique and compared the signaling produced by elderly versus young people. Twenty-four signaling nodes were measured in 12 cell subsets. They confirmed the previous finding of a close association between immune signaling response and age in subsets of CD45RA+ T cells. Fifty-seven nodes showed a pattern of age association, with 51 showing lower responsiveness and 6 showing higher responsiveness in the elderly. Higher responsiveness was observed mostly in B cells, probably in the memory compartment. The authors observed an age association among monocytes, T cells and NK cells, suggesting that the remodeling of immune function virtually splits out throughout all immune cells.

Whiting et al. expanded the measurement of functional immune status by investigating 243 healthy donors aged 40 to 97 years old (80). They described phospho-protein analysis of cytokine signaling, cytokine production, and gene expression from stimulated PBMCs in a stimulation-response manner. This system allowed them to create a public open access platform, encouraging scientists around the world to mine most of the data. The major contribution of this investigation was the evaluation of the functionality of the immune system through different ways, integrating cellular, protein and genomic data at the population level. Age, sex and CMV status may influence immune assays results. For example, phosphoprotein assessment was strongly correlated with CMV status, stimulated cytokine secretion was associated with age, and immune cell phenotype was associated with CMV status and age. Whiting et al. undermined the idea that a single immune system analyte could span immune age, similar to an “immune clock”. They noted a multiple significant association between pairs of analytes. Thereafter, the authors used elastic net regression to establish a model that could predict age. The regression finally listed 14 analytes chosen by the model, including 6 clinical laboratory or morphometric tests, 3 immunophenotyping, cytokines, sex, and CMV status. The model predicted age but overestimated the age of younger participants and underestimated the age of older subjects (80).

Unfortunately, an ideal statistical model that meticulously describes immune system remodeling with age is far from available. Instead, the immune response appears to be best defined as a group of continuous variables closely correlated with each other. Kaczorowski et al. evaluated the immunological phenotype and the functional immune response of 398 individuals and observed that the individuals’ immunotypes are continuously distributed in several healthy individuals (110). Elderly people exhibited greater heterogeneity in their immunotypes than younger people. This finding is consistent with the idea that age alone cannot predict the immune response. How the immune system is remodeled with age depends on an individual’s immunotype.

Even though the previously mentioned studies are endowed with genius, all of them are cross-sectional-based investigations. This imposes severe statistical limitations, as longitudinality becomes an extrapolation. To circumvent this problem, Alpert et al. performed longitudinal screening of various immunological parameters (cellular phenotype, cytokine-stimulation assays and whole blood gene expression) of 135 healthy subjects over 9 years (111). This allowed the authors to follow the cellular and molecular changes that took place in the participants’ immune system year after year to infer a mathematical model that could predict the immune trajectory. They endorsed many previous ratified pieces of confirmed knowledge, for example, that naive CD8 T cells decline with age and the impact of CMV infection on immunosenescence. Interestingly, the data suggested that immune cell phenotype dynamics can be classified into three stages according to convergence at a high-dimensional attractor point. According to the authors, as the immune system ages, the cell dynamics move toward a stable subset´s level, in a steady-state manner. This dynamic could be “slow linear” (e.g. CD85j+ CD8+ T cells), “asymptotic” (e.g. naïve CD4+ T cells) or “fluctuating” (e.g., monocytes), and the cells land to the homeostatic point sequentially. Interestingly, the authors observed that the cytokine response correlated negatively with the immune trajectory model obtained from the collected data. Bringing all these concepts together, the authors defined an IMM-AGE score, which can be valuable in predicting overall survival (111).

## Conclusion

The immune response is under continuous and complex adaptation over time, leading to functional and phenotypic changes in the immune response. The consequence of immune remodeling can be recognized in many clinical features that characterize the older subpopulation. In this context, the evaluation of immunosenescence allows scientists to understand how the immune system ages. It may elucidate new mechanisms by which diseases are established in the elderly. By tracking these mechanisms, potential new targets for aging-related disorders may be revealed. As chronological age does not seem to perfectly reflect the set of molecular and cellular changes that occur with aging, population-level investigations of immune system remodeling allow clinicians to estimate the “immune age” parameter.

The description of immunological age emerges as a new tool to identify the subset of subjects in which unhealthy aging is a matter of time, helping to better individualize clinical management and sort out patients who may benefit from early interventions.

## Author contributions

LC: conception and design, critical review of the literature and data, composition of the manuscript and final approval. LW: critical review of the literature and data, composition of the manuscript and final approval. All authors contributed to the article and approved the submitted version.

## Funding

We thank São Paulo State Research Foundation/FAPESP and Brazilian Society of Endocrinology and Metabology (SBEM) for the investment.

## Conflict of interest

The authors declare that the research was conducted in the absence of any commercial or financial relationships that could be construed as a potential conflict of interest.

## Publisher’s note

All claims expressed in this article are solely those of the authors and do not necessarily represent those of their affiliated organizations, or those of the publisher, the editors and the reviewers. Any product that may be evaluated in this article, or claim that may be made by its manufacturer, is not guaranteed or endorsed by the publisher.
